# (2*E*)-3-(1,3-Benzodioxol-5-yl)-1-(3-bromo-2-thien­yl)prop-2-en-1-one

**DOI:** 10.1107/S1600536810035129

**Published:** 2010-09-04

**Authors:** William T. A. Harrison, C. S. Chidan Kumar, H. S. Yathirajan, B. V. Ashalatha, B. Narayana

**Affiliations:** a Department of Chemistry, University of Aberdeen, Meston Walk, Aberdeen AB24 3UE, Scotland; b Department of Studies in Chemistry, University of Mysore, Manasagangotri, Mysore 570 006, India; c Department of Chemistry, Mangalore University, Mangalagangotri 574 199, India

## Abstract

In the title mol­ecule, C_14_H_9_BrO_3_S, the the prop-2-en-1-one (enone) fragment is close to planar [C—C—C—O = 2.5 (7)°] and it subtends dihedral angles of 12.5 (3) and 5.3 (4)° with respect to the thio­phene and benzene rings, respectively. The dihedral angle between the aromatic ring systems is 12.60 (18)°. Two C—H⋯O inter­actions help to consolidate the non-centrosymmetic crystal packing, which features undulating (100) sheets incorporating *C*(11) and *C*(12) chain motifs.

## Related literature

For related structures, see: Butcher *et al.* (2007 ▶); Harrison *et al.* (2006 ▶, 2007 ▶); Yathirajan *et al.* (2006*a* ▶,*b* ▶,*c* ▶). For background to chalcone derivatives as non-linear optical materials, see: Sarojini *et al.* (2006 ▶). For reference structural data, see: Allen *et al.* (1987 ▶).
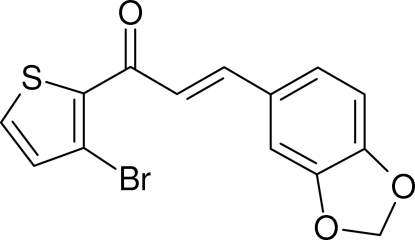


## Experimental

### 

#### Crystal data

C_14_H_9_BrO_3_S
*M* *_r_* = 337.18
Monoclinic, 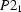

*a* = 4.0013 (3) Å
*b* = 11.0211 (9) Å
*c* = 14.6931 (11) Å
β = 95.781 (2)°
*V* = 644.65 (9) Å^3^
*Z* = 2
Mo *K*α radiation
μ = 3.35 mm^−1^
*T* = 291 K
0.48 × 0.16 × 0.09 mm

#### Data collection

Bruker SMART1000 CCD diffractometer
Absorption correction: multi-scan (*SADABS*; Bruker, 1999 ▶) *T*_min_ = 0.296, *T*_max_ = 0.753
4452 measured reflections
2684 independent reflections
2180 reflections with *I* > 2σ(*I*)
*R*_int_ = 0.032

#### Refinement

*R*[*F* ^2^ > 2σ(*F* ^2^)] = 0.038
*wR*(*F* ^2^) = 0.091
*S* = 0.94
2684 reflections
172 parameters
1 restraint
H-atom parameters constrained
Δρ_max_ = 0.48 e Å^−3^
Δρ_min_ = −0.43 e Å^−3^
Absolute structure: Flack (1983 ▶), 1127 Friedel pairs
Flack parameter: 0.057 (11)

### 

Data collection: *SMART* (Bruker, 1999 ▶); cell refinement: *SAINT* (Bruker, 1999 ▶); data reduction: *SAINT*; program(s) used to solve structure: *SHELXS97* (Sheldrick, 2008 ▶); program(s) used to refine structure: *SHELXL97* (Sheldrick, 2008 ▶); molecular graphics: *ORTEP-3* (Farrugia, 1997 ▶); software used to prepare material for publication: *SHELXL97*.

## Supplementary Material

Crystal structure: contains datablocks I, global. DOI: 10.1107/S1600536810035129/su2204sup1.cif

Structure factors: contains datablocks I. DOI: 10.1107/S1600536810035129/su2204Isup2.hkl

Additional supplementary materials:  crystallographic information; 3D view; checkCIF report

## Figures and Tables

**Table 1:** Hydrogen-bond geometry (Å, °)

*D*—H⋯*A*	*D*—H	H⋯*A*	*D*⋯*A*	*D*—H⋯*A*
C1—H1⋯O2^i^	0.93	2.51	3.420 (6)	167
C14—H14*A*⋯O1^ii^	0.97	2.44	3.400 (6)	171

Symmetry codes: (i) 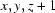; (ii) 
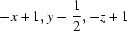
.

**Fig. 1.**
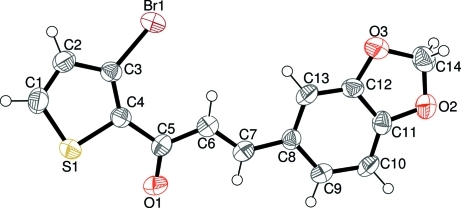
View of the molecular structure of the title molecule showing 50% displacement ellipsoids (arbitrary spheres for the H atoms).

**Fig. 2.**
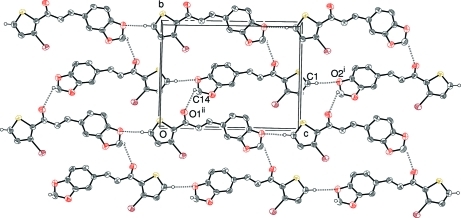
View approximately down [100] of part of a (100) sheet in the crystal structure of the title compound, with C—H···O interactions indicated by double-dashed lines [Symmetry codes: (i) x, y, z + 1; (ii) 1–x, y–1/2, 1–z].

## supplementary crystallographic information

### Comment

The title compound (Fig. 1), was prepared as part of our ongoing synthetic and structural studies of molecules containing thienyl and aromatic rings linked by an enone bridge as possible non-linear optical materials (Sarojini *et al.*, 2006). The crystal structures of (2E)-1-(3-bromo-2-thienyl)-3-(4,5-dimethoxy-2-nitrophenyl)prop-2-en-1-one (Yathirajan *et al.*, 2006*a*), (2E)-1-(3-bromo-2-thienyl)-3-(2,5-dimethoxyphenyl)prop-2-en-1-one (Yathirajan *et al.*, 2006*b*), (2E)-1-(3-bromo-2-thienyl)-3-(4-methoxy-2,3,6-trimethylphenyl)prop-2-en-1-one (Yathirajan *et al.*, 2006*c*), (2E)-1-(3-bromo-2-thienyl)-3-(4-methoxyphenyl)prop-2-en-1-one (Harrison *et al.*, 2006), 1-(3-bromo-2-thienyl)-3-(6-methoxy-2-naphthyl)prop-2-en-1-one (Butcher *et al.*, 2007), (2E)-1-(3-bromo-2-thienyl)-3-(4-nitrophenyl)prop-2-en-1-one (Harrison *et al.*, 2007) have been reported previously.

The C4—C3—Br1 angle in the title molecule of 127.0 (3)° is significantly larger than angle C2—C3—Br1 [118.3 (3)°], perhaps due to steric repulsion between atoms Br1 and H6 (H···Br = 2.75 Å), as also seen in a related structure (Harrison *et al.*, 2007). Br1 is displaced from the C1—C4/S1 ring mean plane by 0.047 (6) Å.

The enone fragment is close to planar [O1—C5—C6—C7 = 2.5 (7)°] and it subtends dihedral angles of 12.5 (3)° and 5.3 (4)°, respectively, with respect to the adjacent thienyl (C1—C4/S1) and benzene (C8—C13) rings. The O and S atoms are in a *syn* conformation [S1—C4—C5—O1 = 11.0 (5)°]. The dihedral angle between the thienyl and benzene ring systems is 12.60 (18)°. The five-membered C11/C12/C14/O2/O3 ring is almost planar (r.m.s. deviation = 0.003 Å) and it subtends a dihedral angle of 0.6 (3)° with ring C8–C13, *i.e.* the rings are statistically co-planar.

In the crystal of the title compound, two weak C—H···O interactions occur (Table 1). The bond involving atom H1 leads to C(12) chains propagating in [001], and that involving atom H14A to zigzag C(11) chains in [010]. Taken together, (100) sheets (Fig. 2) arise, in which unusual *R*^4^_4_(31) loops are apparent.

### Experimental

To 1-(3-Bromo-2-thienyl)ethanone (2.0 g, 0.01 mol) and 1,3-benzodioxole-5-carbaldehyde (1.5 g, 0.01 mol) in 25 ml of methanol, 5 ml of 10% KOH solution was slowly added with stirring at 278 K, and the stirring was continued for 4 h at RT. The solid separated was filtered out and washed with cold methanol. Recrystallization from methanol yielded the pure compound in 90% yield. Pale yellow bar-like crystals of the title compound were obtained by slow evaporation of a solution in acetone (m.p.: 419–421 K).

### Refinement

The H-atoms were geometrically placed (C—H = 0.93–0.97 Å) and refined as riding with *U*_iso_(H) = 1.2*U*_eq_(carrier).

### Crystal data

**Table:**

C_14_H_9_BrO_3_S	*F*(000) = 336
*M**_r_* = 337.18	*D*_x_ = 1.737 Mg m^−^^3^
Monoclinic, *P*2_1_	Mo *K*α radiation, λ = 0.71073 Å
Hall symbol: P 2yb	Cell parameters from 2038 reflections
*a* = 4.0013 (3) Å	θ = 2.3–26.0°
*b* = 11.0211 (9) Å	µ = 3.35 mm^−^^1^
*c* = 14.6931 (11) Å	*T* = 291 K
β = 95.781 (2)°	Bar, pale yellow
*V* = 644.65 (9) Å^3^	0.48 × 0.16 × 0.09 mm
*Z* = 2	

### Data collection

**Table:**

Bruker SMART1000 CCD diffractometer	2684 independent reflections
Radiation source: fine-focus sealed tube	2180 reflections with *I* > 2σ(*I*)
graphite	*R*_int_ = 0.032
ω scans	θ_max_ = 27.5°, θ_min_ = 2.3°
Absorption correction: multi-scan (*SADABS*; Bruker, 1999)	*h* = −5→5
*T*_min_ = 0.296, *T*_max_ = 0.753	*k* = −14→13
4452 measured reflections	*l* = −14→19

### Refinement

**Table:**

Refinement on *F*^2^	Secondary atom site location: difference Fourier map
Least-squares matrix: full	Hydrogen site location: inferred from neighbouring sites
*R*[*F*^2^ > 2σ(*F*^2^)] = 0.038	H-atom parameters constrained
*wR*(*F*^2^) = 0.091	*w* = 1/[σ^2^(*F*_o_^2^) + (0.0549*P*)^2^] where *P* = (*F*_o_^2^ + 2*F*_c_^2^)/3
*S* = 0.94	(Δ/σ)_max_ < 0.001
2684 reflections	Δρ_max_ = 0.48 e Å^−^^3^
172 parameters	Δρ_min_ = −0.43 e Å^−^^3^
1 restraint	Absolute structure: Flack (1983), 1127 Friedel pairs
Primary atom site location: structure-invariant direct methods	Flack parameter: 0.057 (11)

### Special details

**Table:**

Geometry. All e.s.d.'s (except the e.s.d. in the dihedral angle between two l.s. planes) are estimated using the full covariance matrix. The cell e.s.d.'s are taken into account individually in the estimation of e.s.d.'s in distances, angles and torsion angles; correlations between e.s.d.'s in cell parameters are only used when they are defined by crystal symmetry. An approximate (isotropic) treatment of cell e.s.d.'s is used for estimating e.s.d.'s involving l.s. planes.
Refinement. Refinement of *F*^2^ against ALL reflections. The weighted *R*-factor *wR* and goodness of fit *S* are based on *F*^2^, conventional *R*-factors *R* are based on *F*, with *F* set to zero for negative *F*^2^. The threshold expression of *F*^2^ > σ(*F*^2^) is used only for calculating *R*-factors(gt) *etc*. and is not relevant to the choice of reflections for refinement. *R*-factors based on *F*^2^ are statistically about twice as large as those based on *F*, and *R*- factors based on ALL data will be even larger.

### Fractional atomic coordinates and isotropic or equivalent isotropic displacement parameters (Å^2^)

**Table:**

	*x*	*y*	*z*	*U*_iso_*/*U*_eq_	
C1	0.2722 (13)	0.4301 (5)	1.0496 (3)	0.0542 (12)	
H1	0.2566	0.4347	1.1122	0.065*	
C2	0.1769 (12)	0.3322 (4)	0.9984 (3)	0.0454 (10)	
H2	0.0956	0.2606	1.0216	0.054*	
C3	0.2158 (10)	0.3519 (4)	0.9061 (3)	0.0382 (9)	
C4	0.3482 (10)	0.4619 (4)	0.8875 (3)	0.0372 (9)	
C5	0.4449 (11)	0.5265 (4)	0.8055 (3)	0.0417 (9)	
C6	0.3357 (11)	0.4825 (4)	0.7134 (3)	0.0448 (10)	
H6	0.2024	0.4134	0.7060	0.054*	
C7	0.4251 (10)	0.5406 (4)	0.6393 (3)	0.0420 (9)	
H7	0.5671	0.6067	0.6512	0.050*	
C8	0.3315 (10)	0.5148 (4)	0.5429 (3)	0.0375 (9)	
C9	0.4268 (11)	0.5987 (4)	0.4795 (3)	0.0451 (10)	
H9	0.5445	0.6676	0.5007	0.054*	
C10	0.3519 (12)	0.5833 (4)	0.3846 (3)	0.0472 (11)	
H10	0.4155	0.6394	0.3424	0.057*	
C11	0.1795 (11)	0.4802 (4)	0.3591 (3)	0.0419 (10)	
C12	0.0810 (11)	0.3961 (4)	0.4208 (3)	0.0409 (9)	
C13	0.1519 (11)	0.4098 (4)	0.5127 (3)	0.0426 (10)	
H13	0.0850	0.3526	0.5538	0.051*	
C14	−0.0909 (13)	0.3301 (5)	0.2780 (3)	0.0560 (12)	
H14A	0.0216	0.2669	0.2467	0.067*	
H14B	−0.3212	0.3360	0.2506	0.067*	
O1	0.6119 (9)	0.6200 (3)	0.8178 (2)	0.0626 (10)	
O2	0.0773 (10)	0.4431 (3)	0.2707 (2)	0.0610 (9)	
O3	−0.0837 (11)	0.3020 (3)	0.3741 (2)	0.0673 (10)	
S1	0.4233 (3)	0.54359 (10)	0.98785 (7)	0.0466 (3)	
Br1	0.10081 (12)	0.22426 (4)	0.82175 (3)	0.05698 (16)	

### Atomic displacement parameters (Å^2^)

**Table:**

	*U*^11^	*U*^22^	*U*^33^	*U*^12^	*U*^13^	*U*^23^
C1	0.075 (3)	0.052 (3)	0.036 (2)	0.008 (2)	0.010 (2)	0.003 (2)
C2	0.053 (3)	0.038 (2)	0.046 (2)	0.001 (2)	0.013 (2)	0.0039 (18)
C3	0.039 (2)	0.034 (2)	0.041 (2)	0.0020 (17)	0.0000 (17)	0.0001 (17)
C4	0.037 (2)	0.034 (2)	0.039 (2)	0.0027 (17)	−0.0029 (17)	0.0007 (16)
C5	0.044 (2)	0.042 (3)	0.039 (2)	0.0031 (19)	0.0028 (17)	0.0072 (18)
C6	0.051 (2)	0.041 (3)	0.042 (2)	−0.002 (2)	0.0050 (18)	0.0025 (19)
C7	0.045 (2)	0.038 (2)	0.044 (2)	0.0024 (19)	0.0048 (18)	0.0061 (19)
C8	0.043 (2)	0.035 (2)	0.035 (2)	0.0075 (17)	0.0028 (17)	0.0020 (16)
C9	0.054 (3)	0.036 (2)	0.046 (2)	−0.0042 (19)	0.006 (2)	0.0061 (18)
C10	0.057 (3)	0.043 (3)	0.043 (2)	−0.003 (2)	0.011 (2)	0.0155 (19)
C11	0.045 (2)	0.048 (3)	0.033 (2)	0.008 (2)	0.0071 (17)	−0.0012 (18)
C12	0.048 (2)	0.031 (2)	0.045 (2)	0.0013 (18)	0.0104 (18)	−0.0029 (17)
C13	0.051 (3)	0.036 (2)	0.043 (2)	0.0033 (19)	0.0157 (19)	0.0091 (18)
C14	0.063 (3)	0.057 (3)	0.048 (3)	0.004 (2)	0.004 (2)	−0.010 (2)
O1	0.084 (3)	0.047 (2)	0.055 (2)	−0.0276 (18)	−0.0008 (18)	0.0074 (16)
O2	0.088 (3)	0.058 (2)	0.0364 (17)	−0.0052 (19)	0.0062 (16)	0.0002 (15)
O3	0.104 (3)	0.047 (2)	0.051 (2)	−0.019 (2)	0.0081 (19)	−0.0067 (16)
S1	0.0637 (7)	0.0337 (6)	0.0415 (6)	−0.0011 (5)	0.0013 (5)	−0.0049 (4)
Br1	0.0737 (3)	0.0409 (2)	0.0554 (3)	−0.0142 (3)	0.00161 (19)	−0.0071 (3)

### Geometric parameters (Å, °)

**Table:**

C1—C2	1.348 (7)	C8—C9	1.394 (6)
C1—S1	1.692 (5)	C8—C13	1.410 (6)
C1—H1	0.9300	C9—C10	1.406 (6)
C2—C3	1.397 (6)	C9—H9	0.9300
C2—H2	0.9300	C10—C11	1.362 (6)
C3—C4	1.362 (6)	C10—H10	0.9300
C3—Br1	1.901 (4)	C11—C12	1.382 (6)
C4—C5	1.483 (6)	C11—O2	1.383 (5)
C4—S1	1.728 (4)	C12—C13	1.361 (6)
C5—O1	1.232 (6)	C12—O3	1.374 (5)
C5—C6	1.463 (6)	C13—H13	0.9300
C6—C7	1.341 (6)	C14—O2	1.425 (6)
C6—H6	0.9300	C14—O3	1.442 (6)
C7—C8	1.456 (6)	C14—H14A	0.9700
C7—H7	0.9300	C14—H14B	0.9700
			
C2—C1—S1	112.9 (3)	C8—C9—C10	122.5 (4)
C2—C1—H1	123.6	C8—C9—H9	118.7
S1—C1—H1	123.6	C10—C9—H9	118.7
C1—C2—C3	111.3 (4)	C11—C10—C9	115.2 (4)
C1—C2—H2	124.3	C11—C10—H10	122.4
C3—C2—H2	124.3	C9—C10—H10	122.4
C4—C3—C2	114.7 (4)	C10—C11—C12	123.3 (4)
C4—C3—Br1	127.0 (3)	C10—C11—O2	126.8 (4)
C2—C3—Br1	118.3 (3)	C12—C11—O2	109.9 (4)
C3—C4—C5	136.8 (4)	C13—C12—O3	128.4 (4)
C3—C4—S1	109.3 (3)	C13—C12—C11	122.1 (4)
C5—C4—S1	113.9 (3)	O3—C12—C11	109.5 (4)
O1—C5—C6	121.3 (4)	C12—C13—C8	116.9 (4)
O1—C5—C4	117.7 (4)	C12—C13—H13	121.5
C6—C5—C4	121.0 (4)	C8—C13—H13	121.5
C7—C6—C5	120.9 (4)	O2—C14—O3	107.4 (4)
C7—C6—H6	119.5	O2—C14—H14A	110.2
C5—C6—H6	119.5	O3—C14—H14A	110.2
C6—C7—C8	129.2 (4)	O2—C14—H14B	110.2
C6—C7—H7	115.4	O3—C14—H14B	110.2
C8—C7—H7	115.4	H14A—C14—H14B	108.5
C9—C8—C13	119.9 (4)	C11—O2—C14	106.6 (3)
C9—C8—C7	117.4 (4)	C12—O3—C14	106.7 (4)
C13—C8—C7	122.6 (4)	C1—S1—C4	91.8 (2)
			
S1—C1—C2—C3	−2.3 (5)	C9—C10—C11—C12	0.4 (7)
C1—C2—C3—C4	1.8 (6)	C9—C10—C11—O2	−179.5 (4)
C1—C2—C3—Br1	179.3 (3)	C10—C11—C12—C13	−0.4 (7)
C2—C3—C4—C5	178.6 (4)	O2—C11—C12—C13	179.5 (4)
Br1—C3—C4—C5	1.4 (7)	C10—C11—C12—O3	−179.3 (4)
C2—C3—C4—S1	−0.5 (5)	O2—C11—C12—O3	0.6 (5)
Br1—C3—C4—S1	−177.6 (2)	O3—C12—C13—C8	178.8 (4)
C3—C4—C5—O1	−168.1 (5)	C11—C12—C13—C8	0.1 (6)
S1—C4—C5—O1	11.0 (5)	C9—C8—C13—C12	0.2 (6)
C3—C4—C5—C6	13.9 (7)	C7—C8—C13—C12	−179.7 (4)
S1—C4—C5—C6	−167.0 (3)	C10—C11—O2—C14	179.6 (4)
O1—C5—C6—C7	2.5 (7)	C12—C11—O2—C14	−0.3 (5)
C4—C5—C6—C7	−179.6 (4)	O3—C14—O2—C11	0.0 (5)
C5—C6—C7—C8	−177.0 (4)	C13—C12—O3—C14	−179.4 (4)
C6—C7—C8—C9	172.3 (5)	C11—C12—O3—C14	−0.6 (5)
C6—C7—C8—C13	−7.8 (7)	O2—C14—O3—C12	0.4 (5)
C13—C8—C9—C10	−0.2 (7)	C2—C1—S1—C4	1.8 (4)
C7—C8—C9—C10	179.7 (4)	C3—C4—S1—C1	−0.7 (3)
C8—C9—C10—C11	−0.1 (7)	C5—C4—S1—C1	180.0 (3)

### Hydrogen-bond geometry (Å, °)

**Table:**

*D*—H···*A*	*D*—H	H···*A*	*D*···*A*	*D*—H···*A*
C1—H1···O2^i^	0.93	2.51	3.420 (6)	167
C14—H14A···O1^ii^	0.97	2.44	3.400 (6)	171

Symmetry codes: (i) *x*, *y*, *z*+1; (ii) −*x*+1, *y*−1/2, −*z*+1.
